# Characterizing longitudinal blood pressure trajectories in patients at high risk for de novo postpartum hypertension: A randomized controlled trial secondary analysis

**DOI:** 10.1002/pmf2.70012

**Published:** 2025-04-24

**Authors:** Ukachi N. Emeruwa, Minhazur R. Sarker, Natalie Bello, Marni Jacobs, Louise C. Laurent, E. Nicole Teal, Timothy Wen, Russell S. Miller, Cynthia Gyamfi‐Bannerman

**Affiliations:** ^1^ Division of Maternal‐Fetal Medicine, Department of Obstetrics, Gynecology, and Reproductive Sciences University of California San Diego School of Medicine, UC San Diego Health La Jolla CA USA; ^2^ Department of Obstetrics, Gynecology, and Reproductive Sciences University of California San Diego School of Medicine, UC San Diego Health La Jolla CA USA; ^3^ Department of Cardiology, Smidt Heart Institute Cedars‐Sinai Medical Center Los Angeles CA USA; ^4^ Division of Biomedical Informatics, Department of Medicine University of California San Diego School of Medicine, UC San Diego Health La Jolla CA USA; ^5^ Division of Maternal‐Fetal Medicine, Department of Obstetrics and Gynecology Columbia University Irving Medical Center, NewYork‐Presbyterian Hospital New York NY USA

**Keywords:** blood pressure trajectories, blood pressure trends, de novo postpartum hypertension, postpartum blood pressure monitoring, postpartum care, postpartum hypertension, remote blood pressure monitoring

## Abstract

**Introduction:**

De novo postpartum hypertension (dnPPHTN), defined as new‐onset high blood pressure (BP) after delivery in individuals who were normotensive through pregnancy and delivery, accounts for up to two‐thirds of postpartum hypertension cases. Despite its prevalence, there is limited knowledge of BP trends in the postpartum period, hindering opportunities for early detection and timely intervention for dnPPHTN. This study aimed to characterize longitudinal postpartum BP patterns in patients at high risk for dnPPHTN.

**Methods:**

This secondary analysis utilized data from a negative randomized controlled trial (PMID: 38641089) involving 82 normotensive patients at high risk for dnPPHTN, who were randomized to receive either a 5‐day course of 20 mg oral furosemide or placebo starting from postpartum day 1. BPs were monitored every 4‐8 h from delivery to discharge, and subsequently via Bluetooth‐enabled remote monitoring twice daily for 6 weeks. The primary goal of this secondary analysis was to characterize distinct patterns of longitudinal BP trajectories in the placebo group. Secondary goals included exploring differences in early postpartum BP trends between those who developed dnPPHTN and those who remained normotensive, as well as identifying the timing of peak BPs. Trends were assessed graphically using local polynomial regression fitting. Linear mixed‐effects models were used to examine temporal BP trajectories, including random intercepts and slopes, with an interaction term for the time trend and dnPPHTN diagnosis to explore differential impacts.

**Results:**

A total of 40 participants from the placebo arm of the parent trial were included, contributing a total of 2235 postpartum BP measurements. Both systolic BP (SBP) and diastolic BP (DBP) increased until postpartum days 9 and 12, respectively, before subsequently declining. Significant differences in BP trajectories were observed between participants who developed dnPPHTN (*n* = 3; 167 BP readings) and those who remained normotensive (*n* = 37; 2068 BP measurements). Those with dnPPHTN had a significantly steeper rise in SBP preceding the diagnosis, which occurred at a median of 5 days (IQR 5–5.5 days). SBP rose by 1.6 mmHg more per day until its peak at postpartum day 9 in those with dnPPHTN, compared to normotensive participants (*p* < 0.001). DBP rose by 0.3 mmHg more per day and peaked later (postpartum day 14 in those with dnPPHTN vs. day 12 in those who remained normotensive; *p* < 0.001).

**Conclusion:**

Using remote monitoring technology, we characterized distinct postpartum BP trajectories in patients at risk for dnPPHTN, revealing a prolonged rise into the second postpartum week and trends that distinguish physiologic from pathophysiologic BP changes. These findings suggest that extended postpartum BP monitoring may be important for timely identification and intervention in patients developing dnPPHTN.

## 1 INTRODUCTION

Hypertensive disorders of pregnancy (HDP) are a leading cause of maternal morbidity and mortality, contributing to over 50,000 annual deaths worldwide [1]. Studies suggest that more than 80% of pregnancy‐related deaths are likely to be preventable [2, 3]. Postpartum hypertension accounts for 70% of hypertension‐related fatalities, underscoring the critical need for better understanding and management of postpartum hypertension risk factors [4, 5]. Of these postpartum hypertension cases, de novo postpartum hypertension (dnPPHTN), defined as new‐onset high blood pressure after delivery in birthing people who were normotensive through pregnancy and delivery, represents up to two‐thirds [6]. dnPPHTN is particularly challenging because monitoring for individuals who would develop this condition often stops after delivery, making it difficult to determine who needs further monitoring or when new hypertension will develop. Despite this significant impact, the underlying factors contributing to dnPPHTN remain poorly understood.

In the antenatal period, significant progress has been made in reducing hypertension‐related morbidity and mortality [7]. This progress is partly due to a well‐defined understanding of normal versus pathophysiologic blood pressure changes, which facilitates the implementation of timely and appropriate treatment [8]. In contrast, the blood pressure trends in the postpartum period are less understood. Current data on postpartum cardiovascular changes are limited, primarily focusing on patients with antenatal hypertension and scarce data only up to two weeks postpartum on low‐risk normotensive patients [9, 10, 11, 12, 13, 14, 15, 16]. The cessation of routine monitoring for normotensive patients and inconsistent follow‐up practices likely contributed to increased risks of undiagnosed hypertension and related fatalities.

Recent studies in the antenatal period have demonstrated the prognostic value of early pregnancy blood pressure trajectories in identifying individuals at higher risk for HDP before delivery [17]. However, the use of real‐time, prospective longitudinal blood pressure data for risk prediction in the postpartum period has not been explored extensively. This novel, pragmatic approach could offer important insights, particularly for risk‐stratification of patients without a hypertensive diagnosis at delivery. In light of these gaps, our objective was to define longitudinal blood pressure patterns in patients with clinical risk factors for dnPPHTN, addressing an important gap in postpartum care that has implications for timely identification and intervention. By improving our understanding of postpartum blood pressure trends, this work aims to support early identification of patients at risk for developing new‐onset hypertension after delivery.

## 2 MATERIAL AND METHODS

This is a secondary analysis of the placebo arm of the Lasix for the Prevention of de novo Postpartum Hypertension (LAPP) randomized trial of patients at high risk for dnPPHTN [18]. In the parent trial, postpartum patients delivering after 20 weeks’ gestation who had no pre‐delivery diagnosis of hypertension and who had clinical risk factors for new‐onset postpartum hypertension were randomized to a 5‐day course of 20 mg oral furosemide daily or placebo. Risk factors for de novo postpartum hypertension were defined as ≥1 high‐risk criterion or ≥ 2 moderate‐risk criteria according to a prespecified, adapted risk factor algorithm. High‐risk criteria were: pregestational diabetes mellitus, renal disease, autoimmune disease, multifetal gestation, A2 (i.e., medication‐dependent) gestational diabetes mellitus, and history of preeclampsia in a previous pregnancy. Moderate‐risk criteria were primiparity and a BMI ≥ 35 kg/m^2^ at delivery, age ≥ 35 years at delivery, the lived experience of anti‐Black racism, and family history of preeclampsia. This algorithm is based on the American College of Obstetricians and Gynecologists prenatal low‐dose aspirin initiation criteria and adapted according to findings of a pretrial retrospective cohort study that we conducted to identify dnPPHTN risk factors [19, 20].

The primary outcome of the parent trial was mean arterial pressure (MAP) averaged over the 24 h before discharge or the 24 h before antihypertensive therapy initiation, whichever came first. De novo postpartum hypertension was defined as systolic blood pressure (SBP) ≥ 140 mmHg and/or diastolic blood pressure (DBP) ≥ 90 mmHg, following the delivery of a normotensive pregnancy, confirmed by at least two separate measurements ≥ 4 h apart within 6 weeks post‐delivery. Individuals with sustained severe range SBP ≥ 160 mmHg or DBP ≥ 110 mmHg requiring antihypertensive therapy also met criteria for a diagnosis of dnPPHTN. During delivery hospitalization, typically ranging from 24 to 72 h depending on delivery type, postpartum SBP and DBP were recorded every 6 h and more frequently if clinically indicated per institutional protocols. Upon discharge, participants were provided with a Bluetooth‐enabled A&D blood pressure monitor and cuff kit and instructed to measure their blood pressure twice daily (once in the morning and once in the evening) for the remaining 6 weeks postpartum. Blood pressure measurements are automatically transmitted wirelessly from the monitoring device to the electronic medical record. Hypertensive treatment protocols remained unchanged and aligned with standard institutional and national guidelines. Oral antihypertensive medication initiation and adjustments were pragmatic and left to the discretion of the treating physician, based on serial BP measurements and individualized clinical judgment. Additional details of the study have been previously published [18]. The study was approved by the Institutional Review Board of the original study site, an academic urban tertiary care center, and was registered at ClinicalTrials.gov (NCT04752475).

Our secondary analysis was limited to participants in the placebo arm to examine the natural history of postpartum BP trajectories without the potential confounding effects of an intervention, particularly one known to influence fluid balance and hemodynamics. Participants who withdrew prior to assessment of the parent trial's primary outcome were excluded, as they would not have contributed inpatient or outpatient blood pressure measurements to this secondary analysis. Our primary objective was to define the longitudinal blood pressure trajectories in this cohort of patients at high risk for dnPPHTN. Trends in SBP and DBP were assessed graphically using local polynomial regression fitting (LOESS) [21]. We fit linear mixed‐effects models allowing random intercepts and slopes to examine blood pressure trajectories over time (in days). The models were first fit using a maximal mean model for the selection of the covariance structures, assessed by the Akaike information criterion (AIC). We then modeled the mean by comparing polynomial time effects and a linear spline, with the ultimate assumption of a linear spline, best fit at different time points for SBP and DBP.

The cohort was then analyzed in two groups according to their final diagnosis during the parent trial's 6‐week postpartum study period: (1) those who developed dnPPHTN, and (2) those who remained normotensive. Secondary objectives were to explore differences in early postpartum BP trends and identify the peak BPs between those who developed dnPPHTN and those who remained normotensive. An interaction term between the time trend and the diagnosis of dnPPHTN was included in the linear mixed effects models to explore the differential impact of the diagnosis on blood pressure trends.

As an additional subgroup analysis, we used linear mixed‐effects modeling to compare BP trends between patients having at least one high‐risk criteria for dnPPHTN risk and those having only moderate risk criteria. We then further explored trends by combining final diagnosis and risk subgroup, creating four strata: dnPPHTN–high risk, dnPPHTN–moderate risk, normotensive–high risk, and normotensive–moderate risk. Given the small sample sizes within each combination subgroup, results are presented graphically, and statistical modeling was not performed for this analysis.

## 3 RESULTS

From October 2021 to April 2022, 41 participants were enrolled in the placebo arm of the randomized clinical trial. After excluding the one participant who withdrew from the trial during the delivery hospitalization prior to assessment of the primary outcome, we included the remaining 40 participants randomly assigned to placebo, contributing 2235 blood pressure measurements through 6 weeks postpartum. Each participant contributed a mean of 56 BP measurements (range 13 to 98) over the 6 week study period. The demographic and clinical characteristics of the participants included in this analysis are shown in Table 1. Among the placebo arm, 48% (*n* = 19) met eligibility criteria by having at least one high‐risk factor and 52% (*n* = 21) by having at least 2 moderate‐risk factors (Table 2). The median gestational age at delivery was 39.1 weeks [interquartile range (IQR) 38.1, 39.3 weeks] with 55% (*n* = 22) of participants delivering via cesarean. The median BMI at delivery was 30.8 kg/m^2^ [IQR 29.8, 36.1 kg/m^2^]. Low‐dose aspirin for preeclampsia prophylaxis was used by 50% (*n* = 20) of the participants.

**TABLE 1:** Demographic and clinical characteristics of participants at baseline

	Placebo arm (*n* = 40)	De novo postpartum hypertension (*n* = 3)	Normotensive (*n* = 37)	*p* value
**Maternal age at delivery—**median [IQR], years	37.0 [34.0 to 39.0]	39.0 [37.5 to 40.0]	37.0 [34.0 to 39.0]	0.3
**Ethnicity** ^a^—no. (%)				0.7
Hispanic or Latinx	9 (22)	0 (0)	9 (24)	
NOT Hispanic or Latinx	27 (68)	3 (100)	24 (65)	
Unknown/Not reported	4 (10)	0 (0)	4 (11)	
**Race** ^a^—no. (%)				0.8
American Indian/Alaska Native	0 (0)	0 (0)	0 (0)	
Asian	9 (22)	1 (33)	8 (22)	
Black or African American	9 (22)	1 (33)	8 (22)	
Native Hawaiian or Other Pacific Islander	0 (0)	0 (0)	0 (0)	
White	12 (30)	1 (33)	11 (30)	
Multiracial	0 (0)	0 (0)	0 (0)	
Unknown/Not reported	10 (25)	0 (0)	10 (27)	
**Parity**—median [IQR]	1.0 [1.0 to 2.0]	1.0 [0.0 to 1.0]	1.0 [0.0 to 2.0]	> 0.9
**Gestational age at delivery**—median [IQR], weeks	39.1 [38.1 to 39.3]	39.3 [39.3 to 39.3]	39.0 [38.0 to 39.3]	0.3
**Mode of delivery**—no. (%)				0.6
Vaginal	18 (45)	1 (33)	17 (46)	> 0.9
Cesarean	22 (55)	2 (66)	20 (54)	
**Body mass index—**median [IQR], kg/m^2^	30.8 [29.8 to 36.1]	33.8 [29.2 to 38.3]	30.7 [29.8 to 35.9]	> 0.9
**Low‐dose aspirin use during pregnancy**—no. (%)	20 (50)	18 (48)	2 (67)	> 0.9

^a^ Ethnicity and race were self‐reported. Patients of any race could report a Hispanic background. Multiracial is reported as any participant who selected more than one racial category. These data are displayed for the purposes of assessing generalizability and were not used for analysis.

**TABLE 2:** Risk factor distribution among patients with and without de novo postpartum hypertension

	De novo postpartum hypertension (*n* = 3)		Normotensive (*n* = 37)	
**Indication for study eligibility**				
Total with high‐risk criteria (any 1 or more)—no. (%)	2 (67)		17 (46)	
**Distribution of risk factors**				
High‐risk criteria—no. (%)				
Pregestational diabetes mellitus	0 (0)		1 (3)	
Renal disease	0 (0)		0 (0)	
Autoimmune disease	0 (0)		1 (3)	
Multifetal gestation	0 (0)		4 (11)	
Gestational diabetes mellitus, A2	2 (67)		9 (24)	
History of preeclampsia in prior pregnancy	1 (33)		2 (5)	
Moderate‐risk criteria—no. (%)		[Moderate risk only]		[Moderate risk only]
Primiparity	0 (0)	1 (33)	7 (19)	18 (49)
Obesity (BMI ≥ 35 kg/m^2^)	1 (33)	0 (0)	1 (3)	3 (8)
Age ≥ 35 years old	2 (66)	1 (33)	8 (22)	17 (46)
The lived experience of anti‐Black racism	1 (33)	0 (0)	3 (8)	5 (14)
Family history of preeclampsia	0 (0)	0 (0)	0 (0)	1 (3)

*Note*: Numbers (%) in the second and fourth data columns represent the distribution of risk factors in participants with no high‐risk criteria; these were participants who were considered high risk for de novo postpartum hypertension based on the presence of two or more moderate risk criteria.

### 3.1 Patterns of longitudinal blood pressure trajectories

**FIGURE 1:**
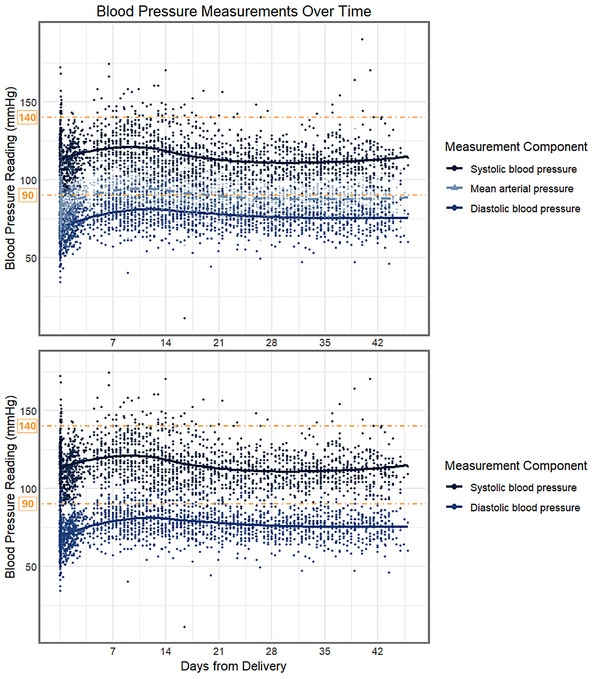
Longitudinal blood pressure measurements over time in the placebo cohort. The longitudinal systolic blood pressure (*top solid line*), diastolic blood pressure (*bottom solid line*), and mean arterial pressure (*middle dashed line in top panel*) trajectories are depicted for the 40 participants in the placebo cohort over the 6‐week postpartum study period. Individual blood pressure measurements recorded for the participants (n = 2235) are represented by the corresponding color points, while smoothed trend lines were estimated using local polynomial regression fitting (LOESS). Threshold indicators (*orange dashed lines*) for 140 mmHg and 90 mmHg are included for reference.

Figure 1 displays the SBP, DBP, and MAP over the first 6 weeks postpartum. The median time to discharge from delivery hospitalization was 2.0 days [IQR 2.0, 3.0 days]. SBP and DBP rose until postpartum days 9 and 12, respectively, after which they declined. The mean peak SBPs and DBPs for the cohort were 120.7 ± 13.4 mmHg and 81.0 ± 10.3 mmHg. As shown in Table 3, from the day of delivery to their respective peaks, the rise in DBP after delivery was twice as steep as the rise in postpartum SBP.

**TABLE 3:** Maximum blood pressures and daily rate of change: Overall cohort and by subgroup

		SBP				DBP			
	Total measurements	Peak (day)	Mean ± SD (mmHg)	Δ to peak (mmHg/day)	Δ after peak (mmHg/day)	Peak (day)	Mean ± SD (mmHg)	Δ to peak (mmHg/day)	Δ after peak (mmHg/day)
Overall cohort	2235	9	120.7 ± 13.4	**0.44**	**−0.61**	12	81.0 ± 10.3	**0.92**	**−0.97**
Postpartum diagnosis									
dnPPHTN	167	9	136.8 ± 12.2	**1.98**	**−2.67**	14	87.9 ± 10.5	**1.13**	**−1.36**
Normotensive	2068	9	119.2 ± 12.1	**0.39**	**−0.52**	12	80.2 ± 9.6	**0.88**	**−0.91**
Risk strata									
High‐Risk	1111	8	121.6 ± 13.2	*(0.62)* ^a^	**−0.59** ^*^	12	81.6 ± 10.4	*(1.05)* ^a^	**−0.74**
Moderate‐Risk Only	1124	10	119.9 ± 13.2	**0.62**	**−0.86**	12	80.3 ± 10.4	**1.05**	**−1.21**

*Note*: Values presented as mean ± SD represent mean blood pressures in the cohort or subgroup corresponding to the day on which the parameter reached its peak. Linear mixed‐effects models were used to examine temporal blood pressure trajectories, incorporating random intercepts and slopes. An interaction term for the time trend and dnPPHTN diagnosis was used to explore differential impacts of postpartum BP diagnosis. A separate model included an interaction term for time trend and the presence of high‐risk criteria to assess differences between risk criteria strata. Δ represents the daily rate of change in blood pressure (mmHg/day) leading up to the peak BP (“Δ to peak”) and following the peak (“Δ after peak”), with bold values indicating statistical significance with *p* < 0.01, except where otherwise noted.

^a^ Δ values in italics indicate that the interaction term for high‐risk criteria was not statistically significant (SBP: 0.43 mmHg/day, *p* = 0.06; DBP: 0.77 mmHg/day, *p* = 0.21), indicating that blood pressure trajectories between high‐risk and moderate‐risk groups did not significantly differ.

* *p* = 0.02.

### 3.2 Trends in blood pressure by postpartum hypertension diagnosis

**FIGURE 2:**
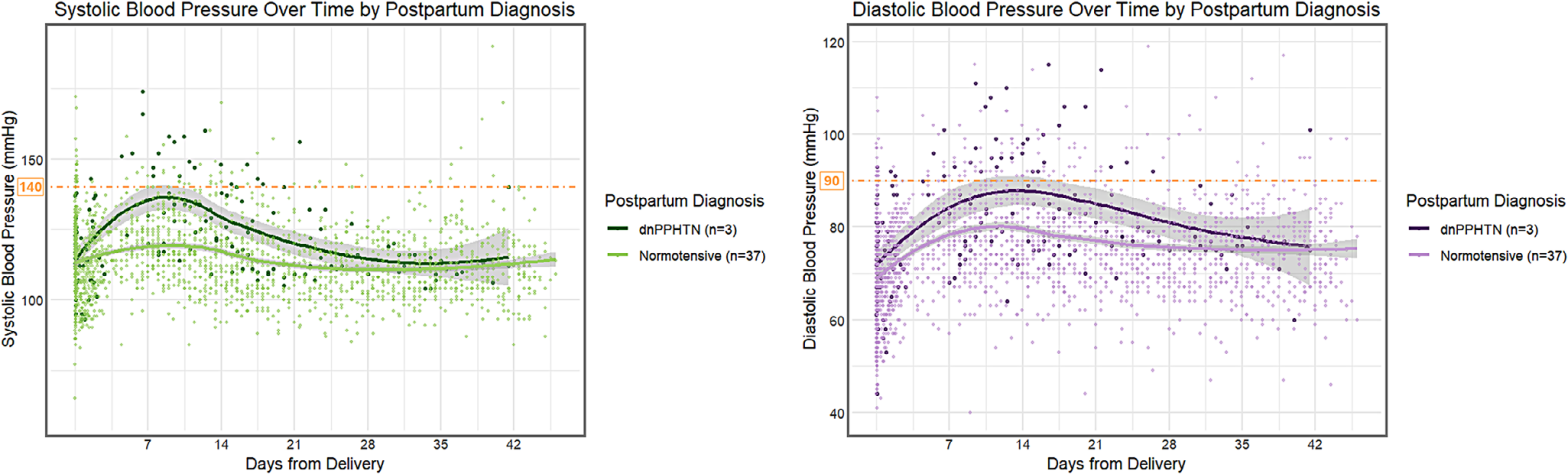
Longitudinal systolic and diastolic pressure measurements over time by postpartum blood pressure diagnosis. The longitudinal systolic (*left panel*) and diastolic (*right panel*) blood pressure trajectories over time are depicted according to the participants' final postpartum blood pressure diagnosis at the conclusion of the 6‐week postpartum study period. In each panel, the top darker curve represents the trend for participants diagnosed with de novo postpartum hypertension (dnPPHTN, n = 3), while the bottom lighter curve represents the trend for those who remained normotensive (n = 37). Individual blood pressure measurements recorded for the participants (n = 167 for dnPPHTN, n = 2068 for normotensive) are represented by the corresponding color points. Smoothed trend lines were estimated using local polynomial regression fitting (LOESS). Threshold indicators (*orange dashed lines*) for 140 mmHg (*left panel*) and 90 mmHg (*right panel*) are included for reference.

Incidence of dnPPHTN was 7.5%. Further details for the participants who developed the dnPPHTN are shown in Table 4.There was a significant difference between SBP and DBP trajectories between participants who developed dnPPHTN (*n* = 3; 167 total BP measurements collected over the 6‐week study period) and those who remained normotensive (*n* = 37; 2068 BP measurements collected over the 6‐week study period) (Figure 2). Those with dnPPHTN had a significantly steeper rise in SBP preceding dnPPHTN diagnosis, which occurred at a median of 5 days [IQR 5, 5.5 days]. SBPs rose by 1.6 mmHg more per day (*p* < 0.001) to a PP day 9 peak in dnPPHTN compared to the PP day 9 peak in normotensive participants. DBP had a more gradual, but nonetheless significantly greater rise than that of the normotensive participants (0.3 mmHg/day more, *p* < 0.001). Notably, in participants who developed dnPPHTN, DBP continued to rise until PP day 14, peaking later than it did in normotensive subjects, for whom DBP peaked at PP day 12 and subsequently declined (Table 3).

**TABLE 4:** Detailed clinical profiles of patients with de novo postpartum hypertension

Participant ID	HIGH‐RISK CRITERIA	MODERATE‐RISK CRITERIA	TOTAL BPS	DNPPHTN DIAGNOSIS (DAY)	ANTI‐HTN INITIATION (DAY)	MGSO_4_ INITIATION (DAY)	PEAK SINGLE SBP (DAY, VALUE)	PEAK DAILY MEAN SBP (DAY, VALUE)	PEAK SINGLE DBP (DAY, VALUE)	PEAK DAILY MEAN DBP (DAY, VALUE)	SMM
6	A2GDM	AMA	47	5	5	5	6 (174 mmHg)	6 (174 mmHg)	14 (98 mmHg)	11 (95 mmHg)	—
18	—	Nulliparity AMA	68	6	6	—	9 (158 mmHg)	9 (158 mmHg)	0 (87 mmHg)	21 (81 mmHg)	Pulmonary edema
43	A2GDM History of preeclampsia in a prior pregnancy	Obesity AMA Experience of anti‐Black racism	52	5	6	5	6 (166 mmHg)	10 (157 mmHg)	16 (115 mmHg)	21 (114 mmHg)	—

Abbreviations: antiHTN, antihypertensive; BP, blood pressure; DBP, diastolic blood pressure; dnPPHTN, de novo postpartum hypertension; MgSO_4_, magnesium sulfate; SBP, systolic blood pressure; SMM, severe maternal morbidity.

All participants diagnosed with dnPPHTN were initiated on antihypertensive therapy, with a median time to initiation of 6.0 days [IQR 5.5, 6.0 days]. Systolic and diastolic trajectories for these participants are shown in Figure S1, illustrating BP patterns in the context of key clinical events.

### 3.3 Trends in blood pressure by risk criteria

**FIGURE 3:**
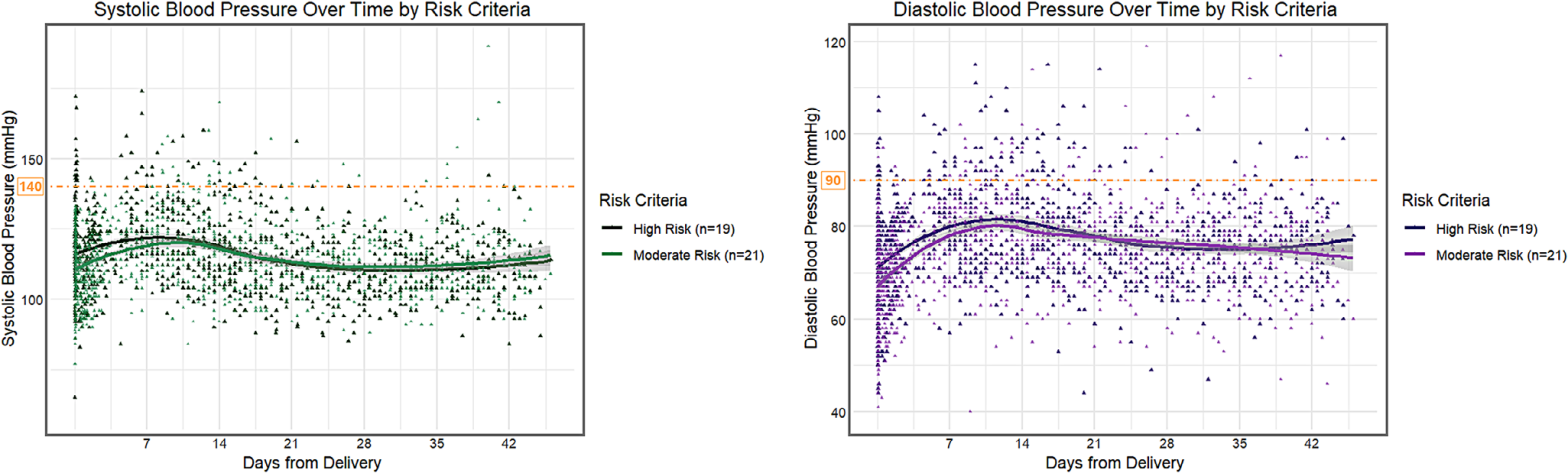
Longitudinal systolic and diastolic pressure measurements over time by de novo postpartum hypertension (dnPPHTN) risk criteria. The longitudinal systolic (*left panel*) and diastolic (*right panel*) blood pressure trajectories over the 6‐week postpartum study period are depicted, stratified by the participants' risk level. In each panel, the darker curve (higher y‐intercept) represents the trend for participants having at least one high‐risk criterion for dnPPHTN risk (n = 19), while the lighter curve (lower y‐intercept) represents those having only moderate risk criteria (n = 21). Individual blood pressure measurements recorded for the participants (n = 1111 for high risk, n = 1124 for moderate risk) are represented by the corresponding color points. Smoothed trend lines were estimated using local polynomial regression fitting (LOESS). Threshold indicators (*orange dashed lines*) for 140 mmHg (*left panel*) and 90 mmHg (*right panel*) are included for reference.

**FIGURE 4:**
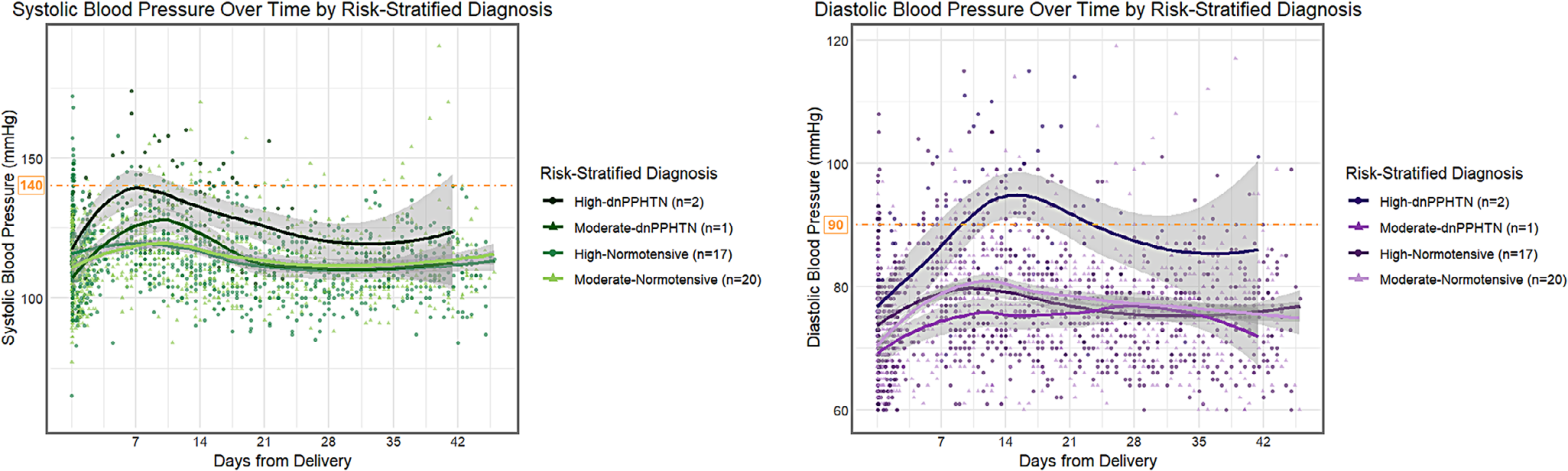
Longitudinal systolic and diastolic pressure measurements over time by risk‐stratified postpartum blood pressure diagnosis. The longitudinal systolic (*left panel*) and diastolic (*right panel*) blood pressure trajectories over the 6‐week postpartum study period are depicted, stratified by the combined risk level‐postpartum blood pressure diagnosis subgrouping. In each panel, the curves (in order from highest to lowest y‐intercept) represent trends for participants in the following groups, respectively: high‐dnPPHTN (n = 2), high‐normotensive (n = 17), moderate‐normotensive (n = 20), and moderate‐dnPPHTN (n = 1). Individual blood pressure measurements recorded for the high‐risk participants (n = 99 for dnPPHTN, n = 1012 for normotensive) are represented by the corresponding color circles; individual blood pressure measurements recorded for the moderate‐risk participants (n = 68 for dnPPHTN, n = 1056 for normotensive) are represented by the corresponding color triangles. Smoothed trend lines were estimated using local polynomial regression fitting (LOESS). Threshold indicators (*orange dashed lines*) for 140 mmHg (*left panel*) and 90 mmHg (*right panel*) are included for reference.

Of the 40 participants included in the analysis, 19 (48%) had at least one high‐risk criterion for dnPPHTN risk. The difference in prevalence of high‐risk criteria between those who developed dnPPHTN and those who remained normotensive (67% vs. 47%, respectively) was not statistically significant (*p* = 0.60). Figure 3 displays the SBP and DBP over the 6‐week postpartum study period, stratified by risk level. SBP reached its peak on PP day 8 for those with at least one high‐risk criterion, compared to PP day 10 for those with only moderate‐risk criteria. There was no difference in the rate of rise in SBP to their respective peaks (Table 4). However, participants with at least one high‐risk criterion had a higher estimated mean day of delivery SBP compared to those with moderate risk criteria alone (estimated mean difference 5.2 mmHg, *p* = 0.048) and also had a slower decline in SBP after reaching their peak (0.5 mmHg/day slower, *p* = 0.02). DBP reached its peak on PP day 12 for both risk groups. Similarly to SBP, there was no difference in the rate of rise in DBP to its peak comparing the two risk groups, but those with at least one high‐risk criterion had a higher estimated mean day of delivery DBP (estimated mean difference 4.2 mmHg, *p* = 0.02) and a slower decline in DBP after reaching the peak (0.5 mmHg/day slower, *p* < 0.001) compared to those with moderate risk criteria alone. Systolic and diastolic trajectories by risk‐stratified postpartum diagnosis are displayed in Figure 4.

## 4 DISCUSSION

In the present study, we defined longitudinal blood pressure trajectories from delivery through 6 weeks postpartum in a cohort of patients with risk factors for HDP, of whom 7.5% developed dnPPHTN. We demonstrated that there may be early pathophysiologic changes in blood pressure preceding dnPPHTN onset, and further, that these changes can be identified through remote monitoring, aiding in timely follow‐up for at‐risk individuals. In the full cohort, both SBP and DBP increased into the second postpartum week, peaking at 10 and 12 days, respectively. While DBP demonstrated a steeper rise overall, there was a significantly greater detectable difference in the rate of increase in SBP for participants who developed dnPPHTN compared to their counterparts who remained normotensive. The greater rates of increase in both SBP and DBP for participants who developed dnPPHTN were detectable prior to diagnosis. While there was no divergence in early PP trends preceding peak BP for participants with high‐risk criteria compared to moderate‐risk criteria alone, participants with high‐risk criteria had higher SBP and DBP levels at delivery and a lagging decline following their respective peaks.

Existing data on the trajectory of postpartum blood pressure changes and the timing of postpartum HDP diagnosis are mixed. There is significant variability depending on the phenotypes represented in the cohort, such as the presence or absence of antenatal hypertension or other comorbidities [9, 10, 11, 12, 13, 14, 15, 16]. This highlights the need for additional data from more comprehensive and diverse populations to better understand these variations. Historically, studies have reported peak blood pressure occurring 4 to 6 days after delivery in patients who were normotensive at delivery [9] and reported an initial decrease in blood pressure after delivery, followed by a rise to hypertensive levels 3 to 6 days postpartum in patients whose pregnancies were complicated by hypertension. However, these studies were limited to the first postpartum week, making it difficult to determine whether such rises continue beyond that period. More contemporary data on normotensive healthy patients monitored through postpartum day 14 reported similar findings (i.e., peak blood pressures within the first week postpartum) but excluded patients with comorbidities that could increase their risk for dnPPHTN, limiting their ability to identify trends in patients at high risk for this phenomenon [12]. Contemporary studies involving patients with antenatal HDP have shown blood pressure patterns ranging from a peak extending into the second postpartum week [5] to a continuous rise from delivery through at least 3 weeks postpartum [16].

The findings from our cohort provide a new and valuable longitudinal perspective compared to existing literature, particularly for a population at risk for an understudied phenomenon that contributes disproportionately to postpartum hypertensive morbidity, i.e., dnPPHTN. Pregnancy is characterized by marked changes in the maternal cardiovascular system, including but not limited to a decrease in systemic vascular resistance and an increase in plasma volume, all of which must return to non‐pregnant physiology after delivery. The prolonged blood pressure rise observed in our study among participants with underlying comorbidities predisposing them to HDP may reflect an underlying impairment in cardiovascular adaptation postpartum, such as persistent vascular dysfunction or a delayed return to baseline cardiovascular physiology. Understanding the mechanisms underlying these prolonged blood pressure elevations can inform the development of targeted therapies, while our findings demonstrate that monitoring blood pressure trends alone may be sufficient for early risk identification.

Our results highlight the clinical importance of extending blood pressure monitoring beyond the first postpartum week. The distinct blood pressure trajectories observed among participants who developed dnPPHTN suggest that early intervention opportunities may be possible if abnormal blood pressure patterns are detected promptly. Traditional monitoring, which often concludes after the first few postpartum days for normotensive patients, may miss important signals in at‐risk patients. Targeted follow‐up for patients exhibiting a continued blood pressure rise beyond the first postpartum week could improve outcomes by facilitating timely interventions to prevent severe maternal morbidity.

A significant strength of our study is the use of remote blood pressure monitoring, which allowed for detailed, longitudinal tracking of blood pressure throughout the postpartum period. This approach provided real‐time data collection over 6 weeks, ensuring comprehensive insight into blood pressure dynamics that could be missed with typical clinic‐based follow‐up. Our focus on normotensive patients at delivery who were at high risk for dnPPHTN is distinct from studies focusing exclusively on those with antenatal hypertension. This unique focus contributes to the literature by identifying early indicators in an enriched risk group that has been underrepresented in postpartum hypertension research. However, our study had some limitations. The sample size, particularly in the dnPPHTN group, was small, and the study was conducted as a secondary analysis from a single center, which limits the generalizability of our findings. Additionally, while our inclusion criteria aimed to identify a high‐risk cohort, the observed incidence of dnPPHTN (7.5%) was lower than expected. While our current risk stratification methods may not fully capture those at highest risk, the distinct BP trajectories identified in this cohort—different from those reported in prior studies—suggest that our approach captured a previously underexplored postpartum physiological phenotype, though the prompt initiation of antihypertensive therapy in all dnPPHTN cases precluded adjustment for treatment effects on BP trajectories and limited our ability to fully characterize the natural history of untreated disease in this subgroup. Finally, we lacked longer‐term follow‐up to determine whether the blood pressure trends observed are predictive of future cardiovascular health. Future studies with larger, more diverse populations and extended follow‐up periods will be needed to validate our findings and broaden their applicability.

## 5 CONCLUSION

In conclusion, our study provides valuable insights into postpartum blood pressure trends, revealing prolonged elevation patterns among patients at risk for dnPPHTN. In contrast to historical data in low‐risk normotensive individuals, which suggests a peak around 4–6 days postpartum, our findings indicate that BP may continue to rise well into the second week after delivery. Thus, extending blood pressure surveillance in the immediate postpartum period beyond existing guidelines and the current standard of care may be important for a risk‐selected cohort of previously normotensive patients. Our findings also suggest greater variability in postpartum BP trajectories than previously recognized, highlighting the need to better characterize diverse postpartum BP phenotypes—beyond those with antenatal hypertension—to optimize risk stratification and intervention timing. Furthermore, we defined distinct trends that may distinguish physiologic from pathophysiologic blood pressure changes in patients at risk for dnPPHTN prior to the occurrence of disease. Real‐time monitoring could be used to identify these early ‘signals’ that foreshadow dnPPHTN, providing opportunities for more personalized postpartum care, timely identification of hypertension, and interventions to prevent severe morbidity.

## CONFLICT OF INTEREST STATEMENT

T.W. serves on the medical advisory board as a consultant for Delfina Care, Inc. The work presented here is not related to his work with the company.

## ETHICS STATEMENT

The parent trial was approved by the Institutional Review Board at Columbia University Irving Medical Center.

## Supporting information



Supporting Information

Supporting Information
