# Supplementary figures and images for: Characterizing longitudinal blood pressure trajectories in patients at high risk for de novo postpartum hypertension: A randomized controlled trial secondary analysis
Source: Pregnancy (Hoboken). Author manuscript; Available in PMC 2025 Sep 16. (PMC12435528; doi:10.1002/pmf2.70012)

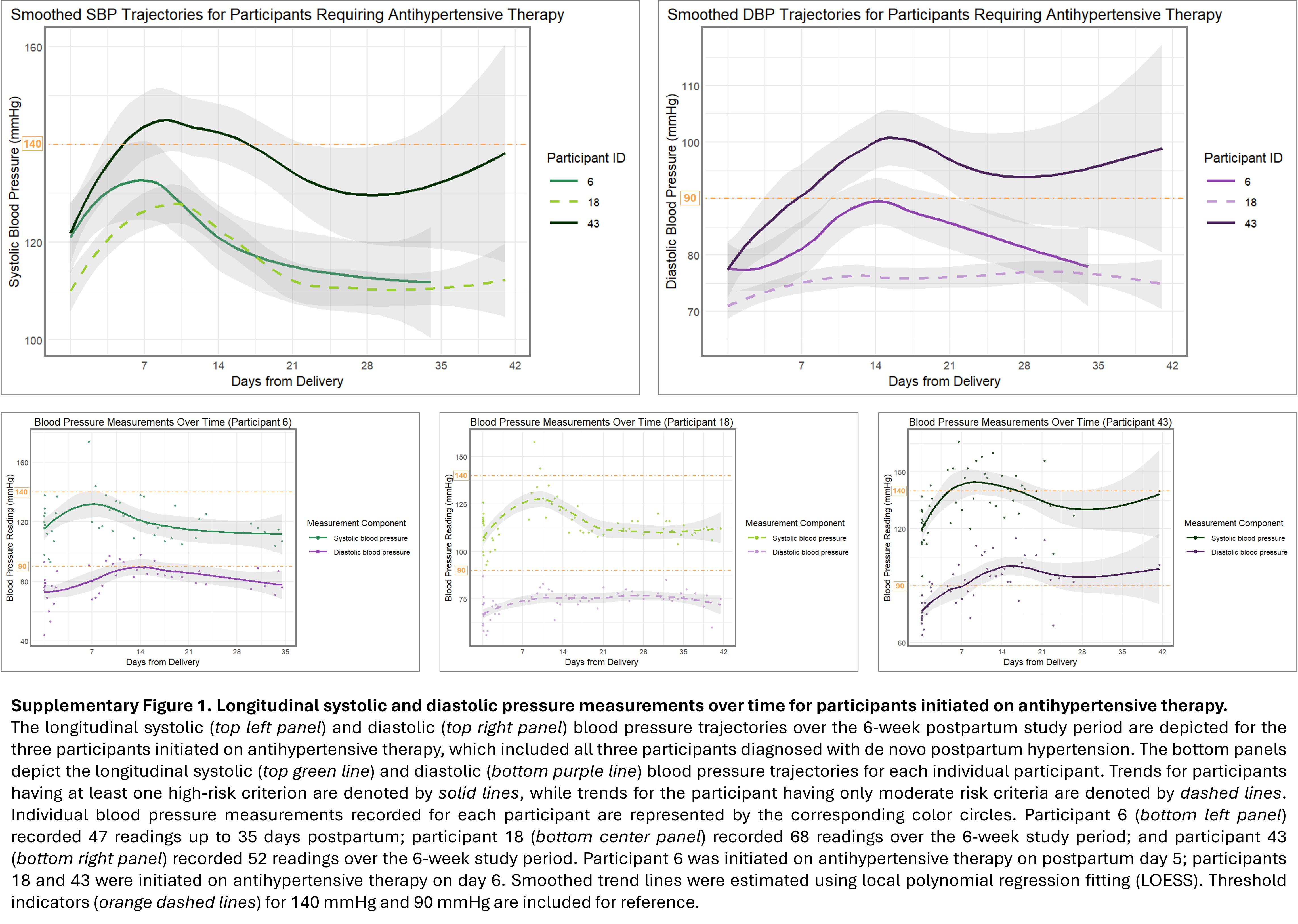

Supplement: Supplemental Figure 1 [file NIHMS2109109-supplement-Supplemental_Figure_1.png]
